# Peer-Assisted Learning in Undergraduate Midwifery Clinical Education: A Qualitative Study on Experiences of Nursing Students From Three Namibian Training Institutions

**DOI:** 10.1177/23779608251328286

**Published:** 2025-04-03

**Authors:** Kristine S Kandingu, Vistolina Nuuyoma

**Affiliations:** 1 School of Nursing and Public Health, 99404 University of Namibia, Windhoek, Namibia

**Keywords:** undergraduate nursing programs, midwifery, nursing education, focus group, clinical settings, midwifery clinical education

## Abstract

**Introduction:**

Peer-assisted learning is widely used in nursing education and is reported to have a positive impact on the students’ learning process. However, students’ experiences of peer-assisted learning from midwifery clinical education in resource-constrained, overcrowded, and small maternity sections are not documented.

**Objective:**

This study was undertaken to explore undergraduate nursing students’ experiences of peer-assisted learning in midwifery clinical education context in Namibia.

**Methods:**

The study was approached from a social constructivism, with explorative, descriptive, and contextual qualitative as a methodological approach. The sample consisted of 32 nursing students from three training institutions, who were conveniently sampled. Data collection was via five focus group discussions, which used a focus group discussion guide, audio recorder, and field notes as research instruments. Data were analyzed using thematic analysis.

**Results:**

Main themes that emerged from thematic analysis are students’ conceptions of peer-assisted learning, benefits, challenges, and suggestions made to improve peer-assisted learning in midwifery clinical education. In thematic area of students’ conceptions of peer-assisted learning, peer teaching tools, engagement, care, and support of peers were recorded as subthemes. The benefits of peer-assisted learning included teamwork, professional identity, a deep approach to learning, communication, coping mechanisms, and socialization. Challenges experienced by students while using peer-assisted learning are learning wrong practices from peers, personality influence, discrimination, labeling, and name calling. Suggestions made by students were formalization and training of students on peer-assisted learning.

**Conclusions:**

Students’ experiences of peer-assisted learning relate to how they understand it as a concept, their interaction with peers, and learning materials. In addition, students made suggestions to improve peer-assisted learning in midwifery clinical practice. These results may be useful in developing peer-assisted frameworks and guiding documents for use in its implementation in midwifery clinical education.

## Introduction

Peer-assisted learning (PAL), or peer learning as known, is a broad concept used for a group of approaches that involve active and interactive mediation of learning through other students who are not professional teachers. Students use teaching and learning interventions to learn from one another without immediate involvement of a teacher (Boud et al., 1999; Topping & Ehly, 2001). Students can be from the same or similar program but not necessarily from the same level or same training institution (Guraya & Abdalla, 2020). Common approaches of PAL are peer assessment, peer monitoring, peer modeling, peer teaching, or tutoring. In addition, team projects, study groups, student-led workshops, student-to-student learning partnerships, and peer feedback sessions are considered as PAL approaches. Irrespective of the approach used, PAL should constitute core elements of communication, collaboration, reflection, self, and peer assessment (Boud et al., 2016). These help students to focus on supporting each other with conceptual understanding, problem-solving and scaffolding from a more competent peer (Shroff et al., 2021). PAL provides students an opportunity for engaged learning experience, timely support services, and a sense of community (Ala et al., 2021), therefore making it suitable for use in both theoretical and practical learning experiences.

## Literature Review

PAL is widely used in health professions education, in which nursing is included, and is reported to have a positive impact on the students’ learning process due to easily accessible of peer tutors (Coliñir et al., 2022). It improves clinical and social skills, increases self-confidence, knowledge, and better understanding of learning materials in health professional students (Sriwigati & Musharyanti, 2022). Moreover, PAL instills a sense of responsibility and dedication among students; improved interpersonal skills and time management skills; reduces passivity in students (Safari et al., 2022); improved students’ self-efficacy (Pålsson et al., 2017); and improved problem-solving skills (Doğan, 2021). Other positive experiences on the use of PAL in midwifery and nursing clinical education are reduced stress, anxiety and other challenges of clinical practice, improved team working skills, which help them prepares for transition to work as a student to qualified staff (Markowski et al., 2021).

In midwifery units, students who use PAL share ideas, thoughts, experience, and knowledge while supporting women in labor and childbirth. There are less involvement of preceptors and promote students to work independently with encouraging feedback from peers (Zwedberg et al., 2021), and it helps improve students’ retention in the program (Neiterman et al., 2023). Furthermore, PAL enhances growth in different competencies and abilities to work together, helping each other to understand and collaborative knowledge creation (Pålsson et al., 2021).

However, use of PAL in clinical education involves challenges of different learning styles which makes it difficult for peers to facilitate learning and lack of consistent applications in many hospitals. In addition, there is no support and structured guidance speaking to implementation of PAL since it is not yet formalized in the clinical learning process (Sriwigati & Musharyanti, 2022). There is limited time in clinical settings, students not ready for PAL, and reluctant to give constructive feedback in fear of compromising their social relationships (Bennett et al., 2015). In some cases, students prefer to focus on attending to their own learning needs and meeting practical requirements instead of helping peers allocated in midwifery units.

Through midwifery clinical education, students are mentored to develop autonomous study practices, in order to cope with stress from practical aspects of the course (Hardy et al., 2014). On the other hand, a considerable amount of the learning that happens in clinical settings at undergraduate level junior years is facilitated informally by senior students (Bennett et al., 2015). Most importantly, learning in midwifery units is opportunistic as it relies on the availability of suitable clients and time for educators to provide proper support to students. As a result, peers become immediate facilitators of learning for other students. Therefore, it is of significance that experiences of students on use of PAL in midwifery units are explored.

Although PAL is a popular topic in medical education literature (ten Cate, 2017), there seems to be limited evidence from middle- and low-income countries and resource-constrained settings, especially in midwifery education contexts. Globally, recent studies conducted on experiences of nursing students on PAL in clinical education focus on pediatric, general wards, and community-based clinical settings (Carey et al., 2018; Galgam et al., 2022; Olsson et al., 2021; Sandvik et al., 2020; Vuckovic & Landgren, 2021); other studies conducted in midwifery clinical education are from developed countries (Doğan, 2021; Hardy et al., 2014; McKellar & Kempster, 2017; McLelland et al., 2013; Neiterman et al., 2023; Zwedberg et al., 2021), while others focus on PAL in laboratory, simulation, and classroom settings which are used for learning midwifery skills (Harahap et al., 2021; Osborne & Othman, 2019; Safari et al., 2022). However, the experiences of nursing students on PAL in midwifery clinical education from contexts of high-income countries may vary from middle- and low-income countries owing to differences in infrastructure, available resources, and technological advancements. Henceforth creating a gap in the availability of evidence from midwifery clinical education in middle- and low-income countries, as well as resource-constrained settings. We conducted this study to explore experiences of nursing students from three training institutions on PAL in undergraduate midwifery clinical education in Namibia.

## Methods

### Study Design

This study was approached from a social constructivism, with the idea that people live in the world of their personal reality, with own way of interpretations of reality (Boyland, 2019). This facilitated the use of qualitative explorative and descriptive designs, therefore helping constructing diverse and complex realities in terms of experiences of students on PAL in clinical midwifery education.

### Research Question

What are the undergraduate nursing students’ experiences of peer-assisted learning in midwifery clinical education context in northeastern Namibia?

### Study Settings

The study was carried out in a maternity section in Kavango East Region, Namibia. The maternity section has a 146-bed capacity, which includes theatre, antenatal care, postnatal wards and two blocks of neonatal care unit. The section receives nursing students from various training institutions in Namibia for their midwifery clinical placements, which varies on durations, usually 2 to 6 weeks of placement. The nursing training in Namibia follows a comprehensive program, consisting of midwifery and nursing courses, which are offered in a four-year university degree, three-year college diploma, and two-year college certificate. Therefore, their clinical practice takes place in maternity, general, and specialized nursing units.

### Study Sample

This study was undertaken with a total of 32 nursing students from three nursing education institutions (NEIs) in Namibia, who participated in the five focus group discussions (FGDs). This sample size was determined by data saturation, which happened when data from new FGDs started repeating what previous groups have stated, and absence of new codes or themes emerging during the analysis of transcripts (Saunders et al., 2018). The inclusion criteria were students who were placed in more than one ward at the maternity section. Excluded were first-year students in both programs because their midwifery curricula focus on antenatal care while fourth level students were excluded because they focus on community midwifery.

### Recruitment and Data Collection Procedures

Participants were recruited while in maternity section for clinical placement. This was done in accordance with convenience sampling (Brink et al., 2018). However, on available students, we sampled for maximum diversity in terms of gender, NEI, program, and levels of study. Each student signed informed consent form before participating in the FGDs. We conducted five FGDs, which consisted of six to seven participants from different NEIs. The first author moderated FGDs, guided the discussions by asking questions, probing and observing group dynamics. All discussions were conducted in English, which is an official language in Namibia, and lasted 45-46 minutes. Data collection was done from May to June 2023. A FGD guide (Table 1), which we designed, was followed, and all FGDs were audio recorded. Piloting was done with a group of students in postnatal ward prior to main data collection to ensure for practical feasibility and identifying flaws (Malmqvist et al., 2019).

**Table 1.** Focus Group Discussion Guide.

**Central question** Tell me about your experience of peer-assisted learning during your midwifery clinical placement in maternity section **Prompts** What do you consider as peer-assisted learning during maternity clinical allocation? What are positive aspects you have experienced on peer-assisted learning while allocated at maternity section? What was challenging or difficult, and why? How did that make you feel? Tell me any suggestion you have on peer-assisted learning in maternity section **Concluding question** Is there anything else you want to add to the conversation regarding peer-assisted learning?

### Data Processing and Analysis

**Figure 1.**
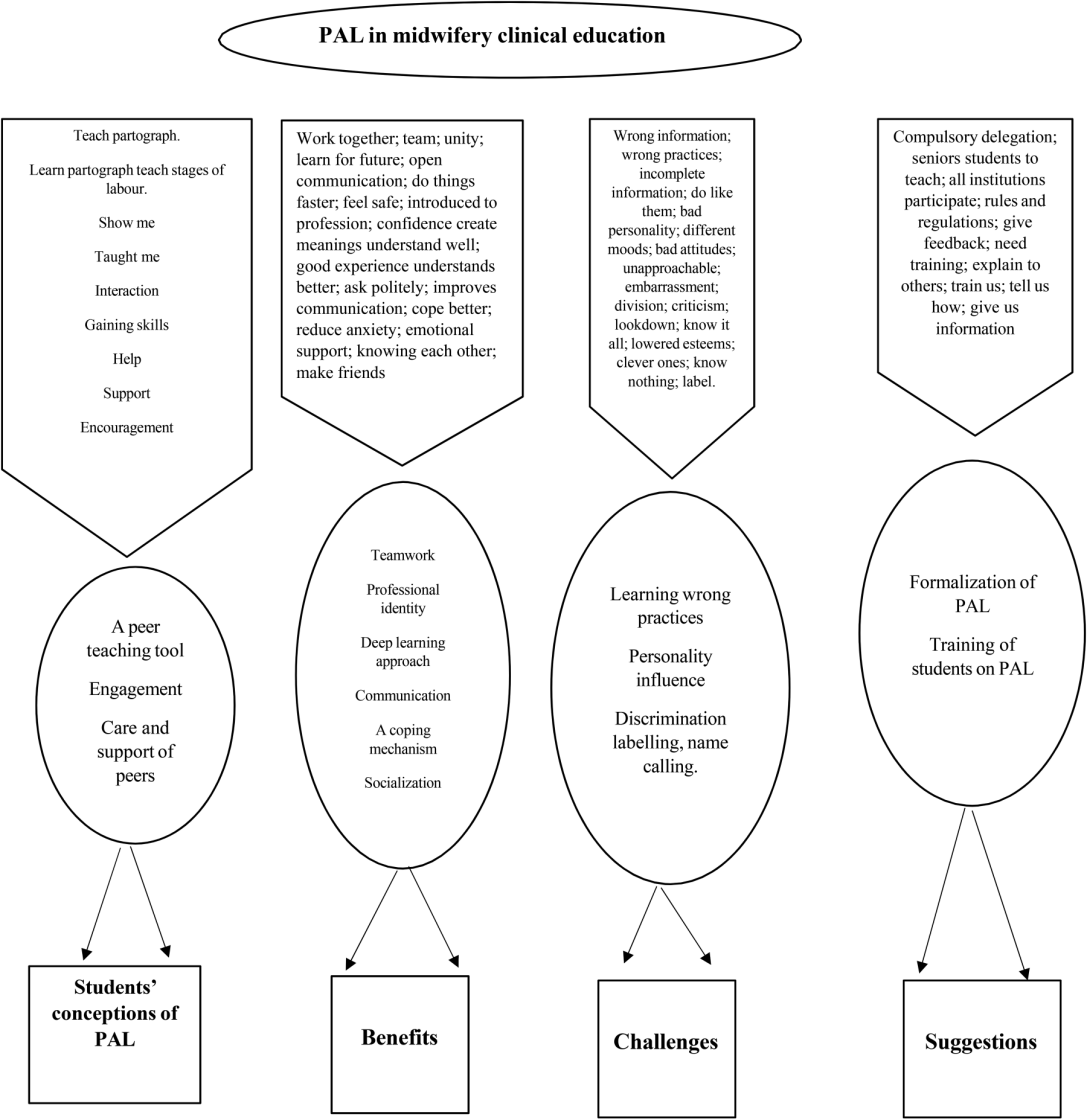
Representation of a coding tree that resulted from thematic analysis.

Audiorecordings from the FGDs were stored in a password-protected mobile device and then transferred to a personal computer. Each audiorecording was transcribed vebatim, followed by data analysis in accordance with thematic analysis (Castleberry & Nolen, 2018), which was done manually by the two researchers. This study was conducted for a degree purpose; therefore, the first author performed analysis and then met the second author for presentation, further analysis, and consensus. The five steps followed in analysis are compiling, disassembling, reassembling, interpreting, and concluding (Castleberry & Nolen, 2018). Most importantly, in reassembling step, we mapped codes and then put them in context with each other. This was to create themes and subthemes. We also conducted a thematic hierarchy in a form of a coding tree (Figure 1) to visualize how themes and subthemes are related and branched out. Lastly, we shared transcripts with participants for validation as a measure to implement member checking.

## Results

### Sample Characteristics

Characteristics of 32 nursing students who participated in the study are presented in Table 2.

**Table 2.** Study Sample Characteristics.

Age	Range	18 to 29 Years
		Number of participants
Gender	Males	13
	Females	19
Nursing education institutions (NEIs)	NEI_1	8
	NEI_2	18
	NEI_3	6
Training program	Bachelor of Nursing Science	15
	Certificate in Nursing and Midwifery Science	17
Levels of study	Second year	24
	Third year	8
Total		32

### Research Question Results

The thematic analysis resulted in 4 main themes and 14 subthemes being extracted, which are presented in Table 3.

**Table 3.** Themes and Subthemes Emerged From Thematic Analysis.

Themes	Subthemes
1. Students’ conceptions of peer-assisted learning	1.1. A peer teaching tool
	1.2. Engagement
	1.3. Care and support of peers
2. Benefits of peer-assisted learning	2.1. Teamwork in midwifery clinical practice
	2.2. Professional identify
	2.3. Deep learning approach
	2.4. Communication
	2.5. A coping mechanism
	2.6. Socialization
3. Challenges of peer-assisted learning	3.1. Learning wrong midwifery practices
	3.2. Personality influence
	3.3. Discrimination, labeling, and name calling
4. Suggestions on peer-assisted learning	4.1. Formalization of peer-assisted learning
	4.2. Training

## Theme 1: Students’ Conceptions of PAL

### A Peer Teaching Tool

PAL is understood as a tool through which peer teaching of practices, knowledge, and skills is facilitated among students while in midwifery clinical practice. They share general information, and some are intended for learning and improving midwifery skills. Knowledge is shared through written notes, textbooks, and internet sources while students interact with each other in midwifery clinical settings. Skills and practices are shared through demonstration and observation.

…my first experience when I came to the maternity unit, I met a student from institution A who taught me how to open a partograph. I learned more about partograph from him, the second day he taught me about stages of labor…. (P2G1)

### Engagement

PAL is understood as a strategy of interacting with other students for the purpose of learning. This is portrayed through interactions that take place while immersed in PAL in clinical practice, which can be either positive or negative aspects.

…the way I understand the word peer assistance learning is an interaction between students that are allocated in the same ward, and the way that they are working together or the way they are communicating be it be it in a good or bad way so just that interaction of students in that particular allocation. (P2G3)

PAL as a form of interactive learning can take place between students from different or same training institutions. It does not consider where students came from, as long as they are in clinical contexts for the purpose of learning midwifery clinical practice.

…let me say learning from your fellow students, be it from the same institution or different institutions…. (P6G3)

### Care and Support of Peers

Although training comes with a lot of pressure, students care for and help others who appear not to be coping while in clinical practice. This is especially considering that in labor unit, some birth procedures can be traumatizing for students. Care from peers is appreciated because students are not always in the accompaniment of clinical instructors and considering shortage of registered nurses and midwives in clinical practice.

I think peer assisted learning is when student nurses care for each other out or even when you see a colleague has been asked do something and then the colleague looks lost then you decide to help that person out” sometimes something traumatizing happens here and we as students take care of each other. (P1G1)

…and every time when I see nurses cutting episiotomy, my heart was not in position but, those students helped me, and they encouraged me and told me a lot of things about maternity. (P3G3)

## Theme 2: Benefits of PAL

### Teamwork in Midwifery Clinical Practice

PAL facilitates teamwork through performing specific actions as a group and in a way that makes practice efficient and effective. This is not only among students but also between students and nursing staff. Participants have observed this especially when there is an emergency in maternity units.

It creates unity between students and staff, because sometimes emergencies happen, other students who are more experienced will be able to say bring this and that, I will say basically it plays important role in the working place. (P5G4)

Participants describe that through PAL, there is work efficiency in clinical practice.

…on the positive part it also encourages teamwork and work will be done easier and faster. (P4G5)

…it also creates team work which create open environment for everyone to work together and share information knowledge…..It also increases team work and things are done faster. (P3G5)

### Professional Identity

Clinical practice is one of the different ways through which students learn what is expected from them in the nursing profession. Through others, students learn what is expected from them, develop a sense of belongingness, and develop professional identity.

the first time I came I worked with fellow students from another institution, they oriented me to things that are found in the ward, they told me if you need help don’t be scared to come ask because they knew it was our first time and I didn’t know what was happening in the ward by that I felt free. I felt safe and really feel I am introduced to my profession; I feel like a midwife. (P5G3)

PAL boosts confidence that is needed by students to build personal characteristics and resilient as a nurse and midwife.

I think aamh working together with other colleagues aah, helps to have confidence it makes you to fit in the environment with others rather than seniors. (P5G1)

I am now more confidence as a health worker, you know….., I have to be strong, just like others, nurses and midwives go through a lot but I am adapting through learning from other students. (P4G4)

### Deep Learning Approach

Nursing students gained in-depth understanding and can easily recall information when subject content is presented by other students in clinical placements, rather than when presented by their lecturers. In addition, students can relate old and new content taught by other students, therefore making new meaning out of them and use for future references.

…also colleagues when they teach us, they refer to topics we covered last year, it is so easier to create own meaning, actually we learn for future. (P7G2)

Positive part of peer assistance learning is you are learning from your fellow student is easier for you to catch up things and also it will be hard for you to forget. You retain information for longer, even able to connect current to previous year's modules. (P3G5)

PAL facilitates introduction of new subject content that is not yet taught through formal training. This facilitates understanding and promotes participation when the lecturer is going to teach a topic that was already covered through PAL.

Positive things that I have learned, you know when I go back to school and the things I have learned from my fellow students. The way my fellow student taught me mechanism of labor, I get to know it better and I am able to explain it in a way that my lecturer will give me good marks on that one. (P1G3)

On the other hand, PAL is experienced as an enabler for “reflection in action” and “reflection on action.”

when a fellow student help me with a learning activity, my though is 100% involved and I asked myself a lot of questions about the activity, while I am performing the activity, and after we are done, I continue to think about the activity and even think of how I can improve my understanding. (P6G4)

### Communication

PAL improves students’ communication skills in a way that they talk politely and portray attributes of good communication while in clinical practice contexts.

Also, the way we are communicating, if I ask a fellow student the response is not harsh but rather in a polite way. They ask politely to repeat if you say something not clear to them, or if they also don’t know, they will tell to ask a next student. (P2G3)

Moreover, students who classify themselves as “shy people” feel encouraged and comfortable to talk while participating in PAL.

It encourages you to talk, it also improves your communication skills like in my case I am actually a shy person…. (P6G1)

### A Coping Mechanism

Students in midwifery clinical practice are under pressure to complete practical logbooks and complete practical hours required. They work overtime to complete pending training-related requirements. At the same time, students are under constant pressure and worries of financial-related issues and unpleased home situations which make them miss formal training sessions by their lecturers. Having other students assisting in academic-related activities reduces anxiety, and they see it as a coping mechanism.

Peer assisted learning I think it's a good thing this can actually reduce anxiety or fear in students, because most students are more open to one another than to their supervisors or let's say their lecturer. So, this means we cope through peers. (P3G2)

Students who were diagnosed with mental illnesses find PAL as a coping mechanism while in clinical practice.

I was diagnosed with bipolar disorder so when I am here with my friends, I get time to talk to them and it helps me, I don’t get stressed. Even when I am scared, or when I hear someone saying something, I join them so yah…. (P6G1)

More support is provided by senior students, therefore helping junior students to cope in clinical practice.

…peer assistance learning help students to get more emotional support and reassurance from the senior students…. (P2G2)

### Socialization

PAL is more than a teaching and learning tool in a way that it facilitates interrelationships among students while in clinical practice. They get to know each other more, learn about different personalities, make new friends, and share more about social life.

…is more about knowing each other as work mates is not about only teaching each other at least you will be able to meet new people with different personalities you will see that this one does not like interacting with others, while this one like socializing so is not only teaching each other's…. (P4G1)

## Theme 3: Challenges of PAL

### Learning Wrong Midwifery Practices

Participants shared concerns about students who want to teach others, but do not have correct knowledge or correct practice of performing a certain procedure. They end up teaching wrong practices, which other students also pass on to others. This was reported as a big challenge because some students may complete their clinical practice without correcting wrong practices.

Sometimes you ask fellow students something, instead of them saying I don’t know, they will tell you a different thing then you start doing the same thing that the student told you about, but later on when the qualified nurse check, she will find something wrongly written in admission book. She will ask who wrote this? Other students will say it's that one, and she will ask who told you that it's written like? This is not supposed to be written like this, oh, some students don’t know but they want to pretend like they know everything…. (P1G3)

Some students are overconfident about their competence and in the process may convince others that they are correct even when they are unsure of the findings on the assessment conducted on clients while in clinical practice. This was reported as a fearful situation as students end up conducting a delivery without supervision of qualified midwives due to reluctance to call for help as they spend time debating on the findings.

There was this other time I was in labor ward, one student from another institution did vaginal examination and she was like no the women is still far while the woman's cervix was 8 cm dilated. We were not all competent and we were afraid to confirm so we were just there in the room we were monitoring and just after a few minutes she delivered there in the room. They were few nurses in the ward we were even afraid to call for help so I was like this student could have gotten us into trouble so some students they are proud they think they know too much. (P5G5)

### Personality Influence

PAL is negatively affected by the personalities of other students. Considering that there is no formal structure followed for PAL, students help others when they feel like doing so. This means students who are more experienced or competent enough to teach others may stay away from assisting due to negative personalities. This is because there is no policy compelling them to help others.

…as students, we are different in term of behaviors and our personalities and the way we associate with others. When you approach someone for help, some don’t give good responses, not and if it's not a good response it will make you to be afraid of asking from someone else because you will think that the person whom you will ask will also respond the same way as the other one responded. (P6G4)

There was this student who was very good with suturing episiotomy, but one day I asked and my dear, she told me a lot of things like I am not your lecturer, I am not obliged to teach anyone braa braa…….wu, she has such a bad personality. (P5G4)

Some students go through different mood swings and respond negatively when others approach them for assistance.

Students come with different moods, sometimes when you ask something they shout I am not here for me, you have a lecturer he supposed to teach you about that I’m not your lecturer and not getting paid for that. (P2G1)

…there is this student that I approached I wanted to ask her something about the partograph, what she answered me is that these things supposed to be taught by your lecturer that answer really hurt me it spoiled my day. (P3G3)

### Discrimination, Labeling, and Name Calling

Discrimination reported was on the basis of training institutions and training program students are registered for. This is because maternity unit receive students from different training institutions and different nursing training program who are allocated for clinical practice at the same time. This different background was reported to make clinical practice difficult for some students due to discriminatory actions and remarks from other students.

Some students when a student from another institution approach them, they will not give the right information because they want to embarrass other students when they make mistake so people will say student from that institution, they don’t know anything. (P4G3)

Challenge of peer leaning is the division of campuses or training centers. So, they end up doing or teaching each other wrong things because they are scared of being criticized by the different institution in the room. This is not good it will also affect the patient, this criticism among student will hold us from asking questions even if don’t know you will be scared of asking questions. (P6G5)

In addition, sometimes students are labeled as not knowing anything or useless simply because they are registered for a specific program or at a specific training institution.

…they might say that you are wrong while you are correct and then they will just be embarrassing you in front of others. Just because you are doing a certificate, it will make you feel kind of like you don’t know anything, and it will just bring your confidence down like you are incompetent. (P1G1)

…they put a name tag on your head, they will be like these students are from ABC institution, they are in second year, they are about to graduate but they don’t know. So, this part actual makes it difficult for other students to approach another student to ask, they will rather stay back not to know the information. (P3G2)

Evidence of name calling was reported from participants. This can be due to the fact that through interaction in a session by another peer, student is perceived not to know or if they are judged by others as overly competent for their level of training when their performance is excellent.

for example a senior student called me to show me how to cannulate a client, the next day, there was a client who needed the drip, the same student was delegated by a sister but she ended up calling me to come do it. I was not able to do it, from there she started telling everyone and started calling me Ms know nothing. (P5G2)

The challenges of peer assisted learning are there are some students who don’t like to be corrected if you correct this student she or he will think you know everything. They even end up calling you names like doctor who who doctor who who. This lowered my esteem, and I don’t have confidence to teach or help other students to learn. You will not even finish your practical book because of name calling. (P4G2)

## Theme 4: Suggestions on PAL

### Formalize PAL in Midwifery Clinical Practice

There should be structures identifying peers who are competent enough to teach and facilitate learning of others and also have open discussions with qualified nurses and midwives on issues emanating from PAL contexts. The students’ delegation and schedule should also incorporate time for PAL.

My suggestion is yes, I understand that maternity ward is a busy ward but, whenever time permits there must be a compulsory delegation for students to have peer discussion. All the students from all institutions must be encouraged to participate and contribute their views on the topic that is discussed. This is because us students we understand each other better than a lecturer thank you. (P7G2)

I feel that it should be implemented as a compulsory thing, because learning from a student or your peer is more like we understand because we are at the same level of understanding as the lecturer or a sister. Maternity ward is very busy ward, sisters will not have time orient and demonstrate on new students if the lecturer or the management of the ward can implement this it should be mandatory that senior student supposed to orientate other students and teach other students when the sisters are not available. (P1G2)

PAL should be formalized due to different types of students in clinical practice and their leaning styles also differs.

I think there should be rules and regulations, and they should be a leader selected from every institution a leader that is there to guide and help others in their journey to develop competences and give them feedback as we are in our final semester is difficult to manage time and study at the same time so I think it will be very good to have a leader who is delegated to teach us feed us with more knowledge. (P5G2)

### Training

Students need to be trained on how to facilitate learning while in clinical practice, considering that they are from different training institutions and levels of study. The maternity unit is also busy, and due to the nature of cases being handled there, it makes the context special.

Maternity ward is busy and sometimes with emergency cases, when nurses are dealing with emergency cases usually, they do not allow students to come near. At least if we know how to do peer teaching and helping others learn, we can keep ourselves busy with other tasks that we need to know in our practical books. The problem is we don’t really know how to go about it. We need even a one-day session by an experienced person who can explain how to help other students learn something. (P6G3)

## Discussion

This study sought to explore how undergraduate nursing students experience PAL in the context of midwifery clinical education, in maternity section. Findings revealed PAL as a peer teaching tool through which students learn from each other. This is done through taking initiative to teach, or in rare cases, they are delegated by qualified nursing personnel. Moreover, our study sees PAL as an engagement tool which facilitates interactions between students while in clinical practice. This also creates a platform for students to display caring attitudes toward each other. This is similar to Sriwigati and Musharyanti (2022) who reported that students developed empathy toward others and patients after participating in PAL. The findings on students’ engagement in PAL were also reported by Pinho et al. (2018). Additionally, our study revealed PAL as a tool through which peers care for each other; this is displayed through identifying others who need support and assist them while in clinical settings. To support these findings, Tamachi et al. (2018) reported a strong sense of support in terms of a wider sociocultural context, while Neiterman et al. (2023) revealed that peers may provide academic, emotional, and instrumental support in the context of midwifery education. However, our current study did not reveal instrumental assistance such as accommodation as part of support students may provide to their peers.

Recent systematic review and meta-analysis studies concluded that PAL has beneficial effects on teaching and learning in health professions education, in which midwifery clinical education is included (Brierley et al., 2022; Markowski et al., 2021; Sriwigati & Musharyanti, 2022; Zhang & Maconochie, 2022). Similarly, our study revealed beneficial aspects of PAL by describing how it promotes teamwork in clinical practice. This is in accordance with Zwedberg et al. (2021) who reported that PAL helps students work well with others and learn collaborative skills, which is vital in management of childbirth. Working together and collaboration due to PAL, which are characteristics of teamwork, were also reported (Markowski et al., 2021; Pålsson et al., 2017).

Professional identity is an important construct in nursing, which is influenced by nursing roles, patient care, health care team, the environment, and nurse's own perceptions of nursing (Rasmussen et al., 2021). In our study, PAL was experienced as an influencing factor on the development of professional identity. This is because, through learning engagement with other students, they learn what is expected from them, develop a sense of belongingness, and admire good behaviors of other nursing students that may help them incline toward a positive professional identity. In most cases, peer educators or facilitators are identified as role models of their peers (Bermingham et al., 2023; Hardy et al., 2014). Moreover, students’ participation in PAL activities helps them to appreciate and develop their future roles and responsibilities as health professionals.

On approaches to learning in clinical practice, our study reported PAL as a promoting factor to deep learning approach. Students who adopt a deep approach to learning engage in reflective practice, critical thinking, and active and self-directed learning and discuss subject matters with others (Alsayed et al., 2021). Stone et al. (2013) discovered that PAL leads to critical thinking and self-confidence in students, while Bermingham et al. (2023) reported that PAL improves students’ comprehension of the course material and supports development of metacognitive skills, which nurture the notion of deep approach to learning. However, Stigmar (2016) documented that there was not enough evidence to support that learning from peers stimulate deep learning approach to learning.

Given the collaborative nature of health professionals’ work, effective communication is required for successful implementation of health care interventions. Advantageously, students in our study revealed PAL led to improved communication among students and with qualified staff members. Improved communication in our study was evidenced by speaking to each other in a polite manner, “shy students” encouraged to ask questions, comfortable to talk in public, and open discussions of cases in maternity section. Equally, improved interpersonal and communication skills as a result of PAL were reported in the literature (Sriwigati & Musharyanti, 2022; Safari et al., 2022; Zwedberg et al., 2021). Although students in our study are from different NEIs, they find PAL a socializing platform and route through which they cope with personal and academic burden. Socialization reported in our study is not only for academic purposes but also for making friends and sharing aspects of their social life. On the same note, Zhang and Maconochie (2022) reported that PAL facilitates development of useful communities of learning clinical skills and knowledge. Students form learning communities via PAL, which enhances their social interactions and makes learning enjoyable (Bermingham et al., 2023). As educational effects of socialization and using PAL as a coping mechanism, Coliñir et al. (2022) reported that PAL reduces drop-out rates, improves academic indicators, and importantly facilitates students to create networks, while Markowski et al. (2021) revealed that PAL helps students to reduce their stress and anxiety levels, as well as cope with challenges of clinical practice, through peer support. Benefits of PAL reported in our study are in support of the essential characteristics of Lave and Wenger's theory of communities of practice (Wenger, 1998), which was used as its philosophical underpinning. The essential characteristics are that members in communities of practice interaction with each other in formal and informal settings share knowledge among members and collaborate among members for the purpose of new knowledge creation, and development of a shared identity is fostered among members in the group (Li et al., 2009). This implies that the practice of PAL has characteristics of a community of practice.

It is evident in our study that students learn wrong midwifery practices from others and go through personality influence, discrimination, labeling, and name calling while practicing PAL. Although their study was not conducted in a midwifery setting, Bhat et al. (2022) reported similar results of interpersonal conflicts among peers, and due to a lack of depth of content knowledge, peer tutors may teach incorrect information, overburden others with irrelevant information which may be inconsistent with curriculum content. However, organizational issues such shortage of learning resources and letting peer tutors be in charge of others for prolonged period without supervision, which were reported by Bhat et al. (2022), were not revealed in our study. Another discordant result reported is that a number of peers opt not to teach others due to the lack of confidence in their knowledge, as preparing and teaching itself can be intimidating (Loda et al., 2020). This was the opposite in our study because students described to be overconfident were willing to share with others, irrespective of their competency level.

Our study revealed suggestions made for improvement. Due to the reason that midwifery sections are busy and attend to unexpected cases, of which some may be emergency, students in our study felt it is necessary to identify competent peers and delegate them for PAL. On the other hand, suggestion was made to train students on the use of PAL, considering that they are from different institutions and different levels of study; it might be challenging for peers to teach and learn from each other without any training. Teaching other students may be a daunting task, especially if not familiar of mechanisms and key aspects to consider in PAL (Stone et al., 2013). It is therefore important for students to be equipped with the know-how of facilitating PAL, or taught how to teach in general, prepare them for leadership, and how to give feedback to others (Burgess et al., 2020; Hardy et al., 2014). Remarkably, a previous study suggested formal integration of PAL in medical student curricula to support improvement of clinical skill performance and development of teaching skills (Brierley et al., 2022). Moreover, integration of PAL into nursing clinical education in addition to preceptorship method may overcome the challenge of limited numbers of nursing staff and at the same time may provide the most effective learning platform (Bahgat & Ahmed, 2021).

## Study Implications

The findings may assist NEIs understand experiences of students on PAL in terms of benefits it offers and its associated challenges. This may help in the development of frameworks and guiding documents on how to implement PAL in a midwifery context. In addition, it may help nurse educators on how they may integrate PAL in routine clinical midwifery teaching and how PAL may help in achieving midwifery clinical education learning outcomes.

## Strength and Limitations

On strength, participants are from three different NEIs, which enhance representativeness and varied experiences of PAL. As a limitation, this study is conducted from a resource-constrained and overcrowded small maternity section, which is a unique context, usually having high patient load and shortage of qualified nursing and midwifery staff, which is different from contexts studied in available literature.

## Conclusions

By exploring the experiences of nursing students on PAL in midwifery context through a lens of communities of practice theory, we revealed students’ conceptions of PAL, benefits, challenges, and suggestions of PAL. This implies that the practice of PAL has characteristics of a community of practices, especially on the benefits revealed by students. These findings are unique considering that the study was conducted from a resource-constrained setting, with a small capacity maternity section, which has a shortage of qualified midwives. They have implications for development of frameworks and implementation guidelines to improve students’ midwifery clinical learning experience. Future researchers may explore experiences and the effect of peer assessment in the context of midwifery clinical education.

## References

[bibr1-23779608251328286] Ala O. G. Yang H. Ala B. K. (2021). Characteristics and comparison of peer-assisted learning interactions among university students in Harbin, China. Social Sciences & Humanities Open, 4(1), 1–8. 10.1016/j.ssaho.2021.100164

[bibr2-23779608251328286] Alsayed S. Alshammari F. Pasay-an E. Dator W. L. (2021). Investigating the learning approaches of students in nursing education. Journal of Taibah University Medical Sciences, 16(1), 43–49. 10.1016/j.jtumed.2020.10.008 33603631 PMC7858010

[bibr3-23779608251328286] Bahgat Z. Ahmed R. (2021). The effect of clinical instructor versus peer assisted learning on students’ knowledge and performance and clinical instructor burnout. Egyptian Journal of Health Care, 12(4), 1497–1506. 10.21608/ejhc.2021.209938

[bibr4-23779608251328286] Bennett D. O’Flynn S. Kelly M. (2015). Peer assisted learning in the clinical setting: An activity systems analysis. Advances in Health Science Education, 20(3), 595–610. 10.1007/s10459-014-9557-x PMC4495258 25269766

[bibr5-23779608251328286] Bermingham N. Boylan F. Ryan B. (2023). The 4C’s of PAL–An evidence-based model for implementing peer assisted learning for mature students. Innovations in Education and Teaching International, 60(3), 401–411. 10.1080/14703297.2022.2050779

[bibr6-23779608251328286] Bhat N. Gurung S. Gupta M. Dhungana N. Thapa R. K. (2022). Enhancing collaborative learning through peer-assisted learning. Journal of Physiological Society of Nepal, 3(1), 4–9. 10.3126/jpsn.v3i1.57762

[bibr7-23779608251328286] Boud D. Cohen R. Sampson J. (1999). Peer learning and assessment. Assessement and Evaluation Higher Education, 24(4), 413–426. 10.1080/0260293990240405

[bibr8-23779608251328286] Boud D. Cohen R. Sampson J. (2016). Peer learning in higher education: Learning from and with each other (pp. 1–184). New York: Routledge.

[bibr9-23779608251328286] Boyland J. R. (2019). A social constructivist approach to the gathering of empirical data. Australian Counselling Research Journal, 13(2), 30–34. www.acrjournal.com.au

[bibr10-23779608251328286] Brierley C. Ellis L. Reid E. R. (2022). Peer-assisted learning in medical education: A systematic review and meta-analysis. Medical Education, 56(4), 365–373. 10.1111/medu.14672 34595769

[bibr11-23779608251328286] Brink H. van der Walt C. van Rensburg G. (2018). Fundamentals of Research Methodology for Health Care Professionals. Juta.

[bibr12-23779608251328286] Burgess A. van Diggele C. Roberts C. Mellis C. (2020). Planning peer assisted learning (PAL) activities in clinical schools. BMC Medical Education, 20(Suppl 2), 1–7. 10.1186/s12909-020-02289-w PMC7712591 33272276

[bibr13-23779608251328286] Carey M. C. Chick A. Kent B. Latour J. M. (2018). An exploration of peer-assisted learning in undergraduate nursing students in paediatric clinical settings: An ethnographic study. Nurse Education Today, 65(February), 212–217. 10.1016/j.nedt.2018.03.014 29604604

[bibr14-23779608251328286] Castleberry A. Nolen A. (2018). Thematic analysis of qualitative research data: Is it as easy as it sounds? Currents in Pharmarcy Teaching and Learning, 10(6), 807–815. 10.1016/j.cptl.2018.03.019 30025784

[bibr15-23779608251328286] Coliñir J. H. Gallardo L. M. Morales D. G. Sanhueza I. C. Yañez O. J. (2022). Characteristics and impacts of peer assisted learning in university studies in health science: A systematic review. Revista Clínica (English Edition), 222(1), 44–53. 10.1016/j.rceng.2021.02.006 34629305

[bibr16-23779608251328286] Doğan N. (2021). Peer supports and problem solving skills of nursing and midwifery students. Journal of Higher Education and Science, 11(1), 150–161. 10.5961/jhes.2021.437

[bibr17-23779608251328286] Galgam F. A. A. R. Mohammed N. E. A. Amin N. M. Nimer M. J. A. (2022). Nursing students’ perception about peer assisted learning and seminar in pediatric nursing at International University of Africa, Sudan. Saudi Journal of Nursing and Health Care, 5(7), 149–155. 10.36348/sjnhc.2022.v05i07.004

[bibr18-23779608251328286] Guraya S. Y. Abdalla M. E. (2020). Determining the effectiveness of peer-assisted learning in medical education: A systematic review and meta-analysis. Journal of Taibah University Medical Sciences, 15(3), 177–184. 10.1016/j.jtumed.2020.05.002 32647511 PMC7336023

[bibr19-23779608251328286] Harahap M. S. Alchalidi A. Baharuddin B. Ramli N. (2021). The effect of peer tutor model on antenatal care skill competencies in learning laboratory skills for students of langsa midwifery study program. Open Access Macedonian Journal of Medical Science, 9(E), 481–484. 10.3889/oamjms.2021.5999

[bibr20-23779608251328286] Hardy E. Hearnden R. Laban A. Northall K. Scurr R. Thompson H. Thouvenel R. Wilson R. (2014). Peer assisted learning in midwifery: A case study. Student Engagement Experience Journal, 3(1), 1–15. 10.7190/seej.v3i1.86

[bibr21-23779608251328286] Li L. C. Grimshaw J. M. Nielsen C. Judd M. Coyte P. C. Graham I. D. (2009). Evolution of Wenger’s concept of community of practice. Implementation Science, 4(1), 1–8. 10.1186/1748-5908-4-11 19250556 PMC2654669

[bibr22-23779608251328286] Loda T. Erschens R. Nikendei C. Zipfel S. Herrmann-Werner A. (2020). Qualitative analysis of cognitive and social congruence in peer-assisted learning – The perspectives of medical students, student tutors and lecturers. Medical Education Online, 25(1), 1–9. 10.1080/10872981.2020.1801306 PMC7482745 32744892

[bibr23-23779608251328286] Malmqvist J. Hellberg K. Möllås G. Rose R. Shevlin M. (2019). Conducting the pilot study: A neglected part of the research process? Methodological findings supporting the importance of piloting in qualitative research studies. International Journal of Qualitative Methods, 18, 1–11. 10.1177/1609406919878341

[bibr24-23779608251328286] Markowski M. Bower H. Essex R. Yearley C. (2021). Peer learning and collaborative placement models in health care: A systematic review and qualitative synthesis of the literature. Journal of Clinical Nursing, 30(11–12), 1519–1541. 10.1111/jocn.15661 33461240

[bibr25-23779608251328286] McKellar L. Kempster C. (2017). We’re all in this together’: Midwifery student peer mentoring. Nurse Education in Practice, 24, 112–117. 10.1016/j.nepr.2015.08.014 26422813

[bibr26-23779608251328286] McLelland G. McKenna L. French J. (2013). Crossing professional barriers with peer-assisted learning: Undergraduate midwifery students teaching undergraduate paramedic students. Nurse Education Today, 33(7), 724–728. 10.1016/j.nedt.2012.10.016 23159008

[bibr27-23779608251328286] Neiterman E. Beggs B. HakemZadeh F. Zeytinoglu I. Geraci J. Plenderleith J. Lobb D. (2023). Can peers improve student retention? Exploring the roles peers play in midwifery education programmes in Canada. Women and Birth, 36(4), 453–459. 10.1016/j.wombi.2023.02.004 36804868

[bibr100-23779608251328286] Olsson, C., Carson, E., Sundin-Andersson, C., Josse-Eklund, A. (2021). All our problems solved? Implementing peer learning in a geriatric hospital setting: A discussion paper. *Nordic Journal of Nursing Research, 41(*2), 61–64. 10.1177/2057158520975307

[bibr28-23779608251328286] Osborne K. Othman M. (2019). Peer assisted learning model to support students’ success in a complex science course. Journal of Nursing Education and Practice, 9(11), 53–62. 10.5430/jnep.v9n11p53

[bibr29-23779608251328286] Pålsson Y. Mårtensson G. Swenne C. L. Ädel E. Engström M. (2017). A peer learning intervention for nursing students in clinical practice education: A quasi-experimental study. Nurse Education Today, 51, 81–87. 10.1016/j.nedt.2017.01.011 28142097

[bibr30-23779608251328286] Pålsson Y. Mårtensson G. Swenne C. L. Mogensen E. Engström M. (2021). First-year nursing students’ collaboration using peer learning during clinical practice education: An observational study. Nurse Education in Practice, 50, 1–6. 10.1016/j.nepr.2020.102946 33310510

[bibr31-23779608251328286] Pinho G. C. Miranda E. P. Tavares M. A. B. Alves D. V. A. de Morais R. X. B. Sobreira T. M. de Almeida S. M. V. (2018). Peer-assisted and team-based learning: A new hybrid strategy for medical education. Revista Brasileira de Educação Médica, 42(3), 162–170. 10.1590/1981-52712015v42n3RB20180042.r2ING

[bibr32-23779608251328286] Rasmussen P. Henderson A. McCallum J. Andrew N. (2021). Professional identity in nursing: A mixed method research study. Nurse Education in Practice, 52, 1–7. 10.1016/j.nepr.2021.103039 33823376

[bibr33-23779608251328286] Safari M. Yazdanpanah B. Yazdanpanah S. Yazdanpanah S. (2022). The experiences and attitudes of student tutors to peer tutoring in the class time of the gynecology and infertility course. Journal of Peer Learning, 15, 66–78. https://ro.uow.edu.au/ajpl/vol15/iss1/6

[bibr34-23779608251328286] Sandvik A. H. Karlsson P. Zetterman A. Eskilsson C. (2020). Nursing students’ experiences of peer learning in a dedicated educational unit in municipal home healthcare: A phenomenological study. Nordic Journal Nursing Research, 41(4), 224–232. 10.1177/2057158520966949

[bibr35-23779608251328286] Saunders B. Sim J. Kingstone T. Baker S. Waterfield J. Bartlam B. Burroughs H. Jinks C. (2018). Saturation in qualitative research: Exploring its conceptualization and operationalization. Quality & Quantity, 52(4), 1893–1907. 10.1007/S11135-017-0574-8 29937585 PMC5993836

[bibr36-23779608251328286] Shroff R. Ting F. Lam W. Cecot T. Yang J. Chan L. (2021). Conceptualization, development and validation of an instrument to measure learners ‘ perceptions of their active learning strategies within an active learning context. International Journal of Educational Methodology, 7(1), 201–223. 10.12973/ijem.7.1.201

[bibr37-23779608251328286] Sriwigati D. Musharyanti L. (2022). Benefits and challenges of peer learning methods in health professional students: A literature review. Bali Medical Journal, 11(3), 1626–1631. 10.15562/bmj.v11i3.3755

[bibr38-23779608251328286] Stigmar M. (2016). Peer-to-peer teaching in higher education: A critical literature review. Mentoring & Tutoring: Partnership in Learning, 24(2), 124–136. 10.1080/13611267.2016.1178963

[bibr39-23779608251328286] Stone R. Cooper S. Cant R. (2013). The value of peer learning in undergraduate nursing education: A systematic review. International Scholarly Research Notices: Nursing, 2013(1), 1–10. 10.1155/2013/930901 PMC3649279 23691355

[bibr40-23779608251328286] Tamachi S. Giles J. A. Dornan T. Hill E. J. R. (2018). You understand that whole big situation they’re in”: Interpretative phenomenological analysis of peer-assisted learning. BMC Medical Education, 18(1), 1–8. 10.1186/s12909-018-1291-2 30107801 PMC6092812

[bibr41-23779608251328286] ten Cate O. (2017). Practice report / Bericht aus der Praxis: Peer teaching: From method to philosophy. Zeitschrift für Evidenz, Fortbildung und Qualität im Gesundheitswesen, 127, 85–87. 10.1016/j.zefq.2017.10.005 29128431

[bibr42-23779608251328286] Topping K. J. Ehly S. W. (2001). Peer assisted learning: A framework for consultation. Journal of Educational Psychology Consultation, 12(2), 113–132. 10.1207/S1532768XJEPC1202_03

[bibr43-23779608251328286] Vuckovic V. Landgren K. (2021). Peer learning in clinical placements in psychiatry for undergraduate nursing students: Preceptors and students’ perspective. Nursing Open, 8(1), 54–62. 10.1002/nop2.602 33318812 PMC7729660

[bibr44-23779608251328286] Wenger E. (1998). Communities of practice: Learning, meaning, and identity (pp. 1–336). Cambridge: Cambridge University Press. https://psycnet.apa.org/doi/10.1017/CBO9780511803932

[bibr45-23779608251328286] Zhang Y. Maconochie M. (2022). A meta-analysis of peer-assisted learning on examination performance in clinical knowledge and skills education. BMC Medical Education, 22(1), 1–14. 10.1186/s12909-022-03183-3 35248051 PMC8897892

[bibr46-23779608251328286] Zwedberg S. Alnervik M. Barimani M. (2021). Student midwives’ perception of peer learning during their clinical practice in an obstetric unit: A qualitative study. Nurse Education Today, 99(January), 1–6. 10.1016/j.nedt.2021.104785 33524896

